# Validation of the Adaptive Danish Sentence Test (DAST): Normative Data from a Template-Based, Linguistically Rich Sentence-in-Noise Test

**DOI:** 10.3390/audiolres16030075

**Published:** 2026-05-19

**Authors:** Abigail Anne Kressner, Kirsten Maria Jensen-Rico, Anja Kofoed Pedersen, Lars Bramsløw, Brent Kirkwood

**Affiliations:** 1 Clinical and Technical Audiology, Hearing Systems Section, Department of Health Technology, Technical University of Denmark, 2800 Kongens Lyngby, Denmark; 2 Copenhagen Hearing & Balance Centre, Department of Otorhinolaryngology, Head and Neck Surgery & Audiology, Rigshospitalet, 2100 Copenhagen, Denmark; 3 WS Audiology, 3540 Lynge, Denmark; 4 Demant, 2765 Smørum, Denmark; 5 GN Hearing, 2750 Ballerup, Denmark

**Keywords:** speech-in-noise, speech intelligibility, speech reception threshold

## Abstract

**Background/Objectives**: This study describes the development and validation of the Danish Sentence Test (DAST), a Danish-language, adaptive speech-in-noise test constructed from a linguistically balanced corpus using a template-based method. This approach enables controlled linguistic variation while maintaining lexical consistency and may serve as a model for developing similar speech materials in other languages. **Methods**: Sentences spoken by one female talker from the DAST corpus were sorted into 44 balanced lists of 20 sentences using a psychometric optimization procedure. Speech reception thresholds (SRTs) were measured in 20 normal-hearing participants using headphone playback with speech-shaped noise. **Results**: Across the 44 sentence lists, the mean SRT was −5.3 dB SNR, with list means within ±0.5 dB of the grand average under the tested configuration. The average within-subject standard deviation was 0.7 dB, and the grand-average psychometric slope was 18.5%/dB. A statistically significant within-session training effect of approximately 0.02 dB per measurement. **Conclusions**: This study provides normative speech reception threshold (SRT) data for the adaptive Danish Sentence Test (DAST) in normal-hearing listeners under a defined headphone-based speech-in-noise paradigm and demonstrates that the resulting sentence lists yield comparable performance across lists. The template-based construction and optimization approach offers a framework for developing linguistically rich sentence-in-noise tests in other languages.

## 1. Introduction

Speech tests are widely used in audiological clinics, research laboratories, and industry alike to assess an individual’s ability to understand speech [1,2,3]. Speech tests are inherently language-specific, and for the Danish language, there are a plethora of tests that exist already, each with their own specific purpose. Many clinically oriented tests are based on word- and phoneme-based speech corpora, including Dantale I [4], the Northwestern University Auditory Test No. 6 (NU-6) [5], and the Consonant Nucleus Consonant (CNC) monosyllabic words [6]. While word- and phoneme-based speech tests assess important aspects of speech perception, they are less suited for assessing a listener’s ability to follow natural forms of communication. Because each word is often presented in isolation, the words or phonemes often do not include many of the characteristics of natural speech, and as a result of this, it becomes difficult to assess more modern hearing aids, as the underlying adaptive algorithms do not necessarily work as intended in such unnatural listening scenarios (e.g., compression and noise reduction). Investigations of speech understanding, therefore, often employ sentence-based speech material instead.

One example of a sentence-based test is the matrix sentence test [7,8,9], which is a type of test available in many languages around the world. Every list in the corpora consists of five-word sentences with the same syntactical structure. The structure of this type of material in general gives a level of control that can be very advantageous in many applications. The fixed syntactical structure of a matrix sentence test, however, increases predictability, which can lead to a large learning effect and relatively low speech reception thresholds (SRTs) [10]. An alternative approach to matrix sentence testing is the hearing in noise test (HINT), which is a speech-in-noise test consisting of sentences that represent the pronunciation and characteristics of conversational speech, namely with varying syntax, meaningful context and an open set of vocabulary [11]. This test has been made into an international standard and is also available in a large number of languages around the world.

Investigations of speech in Denmark where linguistic variation is important have to date primarily been limited to the Danish HINT [12]. A typical investigation that involves speech-in-noise testing can easily demand collection of more than 10 data points in a session, and this has not been possible in practice without repetition of the sentence lists in the Danish HINT. This is because the test consists of only 200 sentences, divided into 10 lists, which for reference is very little in comparison to popular English-language speech corpora like the Texas Instruments/Massachusetts Institute of Technology (TIMIT) database [13], the IEEE corpus of sentences [14], and the Bamford-Kowal-Bench (BKB)-like sentences [15] that contain 6300 sentences, 720 sentences, and 1280 sentences, respectively. One commonality between all these larger, English-language corpora is that they provide substantially more linguistic variation than existing Danish-language speech corpora, by including, for example, a large number of speakers spanning a range of dialects, by containing sentences that tend to be longer in duration, or simply by including many more sentences to increase the range of linguistic variation and amount of vocabulary. However, the increased variation that exists in each of these corpora can come, at least in some applications, with the downside of less control.

Thus, a new approach for constructing a speech corpus was proposed in order to facilitate linguistic variation in a controlled way [16]. In short, sentences are constructed using a “template-based” method just as previous corpora have done (e.g., matrix sentence tests). However, instead of basing the material on one relatively simple syntactical structure, ten different templates are employed, each with a relatively complex, but flexible sentence structure. Moreover, each template contains specific placeholders for defining exactly three keywords. Sentences are then created from these templates using words from a list of the most frequently used words in the Danish language as the keywords. Thereafter, additional words are filled in to form a coherent and natural sentence that is linguistically complex. Importantly, we have shown that use of this template method does not induce systematic differences in the psychoacoustic properties of the sentences associated with each template [17].

The aim of the present study was to develop and validate the adaptive Danish Sentence Test (DAST), a Danish-language, sentence-based speech-in-noise test designed to provide substantially more material than existing Danish tests and thereby enable extended measurements without sentence repetition. Validation in this study was achieved by sorting the material into equivalent sentence lists and establishing normative speech-in-noise performance data under a single, well-defined experimental configuration involving headphone-based playback and stationary speech-shaped noise in young, normal-hearing listeners. In this context, validation refers to the establishment of normative reference data and the demonstration of comparable performance across sentence lists under these test conditions, rather than a comprehensive evaluation across listener populations, playback setups, or scoring paradigms. Although developed for Danish, the template-based construction and optimization approach is presented as a general methodological framework that may inspire the development of linguistically rich sentence-in-noise tests in other languages.

## 2. Materials and Methods

### 2.1. Speech Material

Speech material was selected from the DAST corpus, (http://doi.org/10.11583/DTU.24058110) which is a corpus of audio and audio-visual recordings of 1200 Danish sentences spoken by four different talkers. Example sentences, their keywords, and their respective template types are given in Table 1. Each of these sentences have previously been assessed on (1) how natural they are on a 7-point Likert scale and (2) whether they evoke a feeling of emotional discomfort or negativeness. The sentences were also assessed on the quality of the pronunciation, and whether the sound and video quality were sufficient for use in a speech-in-noise test with audio or audio-visual playback, respectively. Additional information about the development of the sentences and their linguistic evaluation can be found in Kressner et al. [16]. In addition to this, all 1200 sentences from one of the female talkers (F1) have been psychoacoustically characterized [17]. Each psychometric function is described by three parameters: the inflection point, the slope of the curve at the inflection point, and the mean squared error (MSE) of the fitted function. These data were collectively used to filter out problematic sentences.

Cutoffs for the discomfort and naturalness data were heuristically chosen to remove only the worst sentences. Cutoffs for the psychometric function parameters were identified as elements more than 1.5 interquartile ranges above the upper quartile (75 percent) or below the lower quartile (25 percent); however, the lower cutoff for the slope was adjusted to be zero rather than negative, and the lower cutoff for MSE was removed altogether. Thus, from the 1200 sentences available, sentences that satisfied the following criteria were retained, resulting in 977 sentences after filtering:

- Of sufficient quality for both audio and audio-visual playback.
- Evoked a feeling of discomfort in less than 25% of survey participants.
- Mean naturalness score of at least 4.5.
- Inflection point that was greater than −9.2 dB and less than −0.5 dB.
- Slope that was greater than 0%/dB and less than 33.5%/dB.
- MSE that was greater than 897%^2^.

To normalize the intelligibility of each sentence, the level of each sentence was increased or decreased by an offset amount that was equivalent to the difference between the inflection point of the individual sentence and the mean inflection point across the sentences. To be clear, the digital level of each of the wav files in the corpus remains at −26 dB FS. However, we apply an RMS adjustment to each sentence immediately before playback via custom-built software. This adjustment effectively shifts the signal-to-noise ratio (SNR) at which each curve equals 50% so that the inflection point for each individual sentence matches that of the mean inflection point. This sentence normalization procedure has been applied in many tests before, the consequences of which are well-described [8].

The mean inflection of the remaining 977 sentences was −4.8 dB. Sentences with an inflection point more than 3 dB from this mean were also filtered out, effectively limiting the magnitude of the offsets that would eventually be applied to 3 dB, as the mean of the inflection points in the final set of sentences did not substantially change from this point on. An additional 52 sentences were filtered out due to this criterion, resulting in 925 sentences for the optimization. However, not all templates were represented equally among the sentences that remained. There were though at least 88 instances of each template type. Since the sentences were organized into equivalent lists of 20 sentences each, it was possible to include two sentences of each template type. Under these constraints, a maximum of 44 lists could be created.

Sorting of the sentences was treated as an optimization problem with a cost function that quantified the amount of variation in the psychometric properties between the lists. The sentences were pseudo-randomly sorted into lists 1000 times, each time generating a different set of lists. To sort the sentences into lists in each iteration, a pseudo-randomization method was applied as follows. The sentences corresponding to each template were first arranged in increasing order according to their offset values. The sentences were then paired to balance out the extremes. Pairing began with the two middle sentences (i.e., those with the closest offset values, typically around 0 dB), and expanded outward, progressively matching sentences with increasingly extreme offset values. The process concluded with a pair of sentences having the most extreme offset values (i.e., close to −3 dB and +3 dB, respectively). The pairs of sentences for each template type could then be randomly distributed across lists, so that extreme values of offsets were balanced both across and within lists.

After each iteration of sorting, the mean psychometric function was updated based on the chosen set of 880 sentences (i.e., 44 lists of 20 sentences), and the sentence-specific offset values were recalculated. Thereafter, the mean of the slope and offset were calculated for each list. The cost function was then computed as a weighted sum of the standard deviation of these means with equal weights given to the slope and offset. The set of lists resulting in the lowest cost, meaning the smallest variation in the psychometric properties across lists, was chosen as the final set of lists. Finally, a CSV file was exported with the defined lists, containing list numbers with their corresponding sentence number and offset value for sentence normalization, which is available for download (https://doi.org/10.11583/DTU.24058110). The first list of the final set is shown in Table 1.

### 2.2. Participants

Twenty normal-hearing (NH) adults (13 females and 7 males) with Danish as their native language participated in the intelligibility assessment. The ages ranged between 20 and 39 yrs with a mean age of 24.8 yr (standard deviation 3.9 yr). The participants underwent audiometric testing to ensure hearing thresholds better than 20 dB HL from 250 Hz to 8 kHz in both ears. All participants read and signed an informed consent to participate in this study and were offered monetary compensation for their transportation expenses. The procedure was approved by the Science-Ethics Committee for the Capital Region of Denmark (reference H-16036391).

### 2.3. Procedure

Participants were seated in a sound attenuated test booth, and their hearing thresholds were measured via an Interacoustics AC40 audiometer (Interacoustics A/S, Middelfart, Denmark) and Sennheiser HDA200 audiometric headphones (Sennheiser, Wedemark, Germany). Thereafter, participants listened to speech material through Sennheiser HDA200 headphones (Sennheiser, Wedemark, Germany) connected to a desktop computer via an RME Fireface UC USB sound card (RME, Haimhausen, Germany). Minimum phase equalization filters were applied to all stimuli to ensure a flat frequency response from the headphones when measured with a GRAS 43AA ear simulator kit (GRAS Sound & Vibration, Holte, Denmark). The tester was seated outside of the booth in a control room and had a microphone to communicate with the participant. Purpose-built software was created and modified in order to administer the test adaptively.

Background noise was presented at a fixed level of 70 dB (A) throughout the duration of each list. The noise was a stationary noise shaped to have the same long-term average spectrum of the talker [17]. The speech level for the first sentence in each list was 0 dB SNR. In a manner inspired by the HINT guidelines outlined in the user manual (Bio-logic Systems Corp., Mundelein, IL, USA), if the first sentence was not repeated completely correctly, it was presented again with incremental increases of 5 dB until repeated correctly. It is also recommended to restart measurements if perseveration is suspected or if the first four sentences in a list are repeated correctly; however, neither situation occurred in the present study.

The level of the speech was adjusted thereafter in each presentation following an adaptive methodology inspired by Keidser et al. [18], where the SNR is adjusted in varying step sizes. During the first phase, the step size was 5 dB for at least 4 sentences and until the first reversal. For the second phase, the step size was 2 dB for at least 4 additional sentences and until the standard error (SE), computed from the presentations only in this phase, was less than 1 dB. For the third and final phase (which was not always reached), the step size was 1 dB. The number of sentences presented in each phase was therefore variable and depended on convergence of the adaptive track but never exceeded the 20 sentences available in a list. The speech level was adjusted at each presentation until all the sentences in the list were completed. The SRT was calculated as the average SNR over only the sentences in the second and third phases. The SE of the measurement was similarly calculated from only the sentences presented in the second and third phases. Note that the presentation levels used to calculate the SRT and SE are the unadjusted levels (i.e., the presentation levels before the sentence-specific offsets were applied).

The participants were asked to listen to the audio stimuli and to repeat back the sentence, or any of the words heard. From the control room, the tester scored the sentence based on the response given using keyword scoring and a set of rules based on spoken language, which have been previously described [17]. In short, only variations that appear naturally in spoken language were accepted (e.g., variations due to dialect or due to assimilation). The participants were not made aware of the focus being on keywords. To familiarize the participants with the task, they listened to two lists prior to the test session. The training lists were randomly chosen for each participant from the set of lists that would not be presented during the test session for that participant. After the training phase, the participants were presented with one-half of the lists. To pseudo-balance the lists across listeners, a random permutation of the lists was created for each set of two listeners. This way, in each consecutive set of two subjects, all the lists were evaluated. With this setup, a total of 20 participants were needed to obtain 10 scores for each list. All testing and scoring were conducted by the same trained tester across all participants. The duration of each participant’s visit was typically 2–2.5 h.

### 2.4. Analysis

Analyses were carried out using MATLAB 25.2.0.3123386 (R2025b) Update 3 (Mathworks, Natick, MA, USA). For the validation of list equivalence, mean SRTs and standard deviations (SDs) are reported for each list. Normalized SRTs are also reported to assess the variation across lists from the grand average. To investigate the within-subject variability, mean SRTs across lists, as well as the within-subject SD, are reported for each participant. Thereafter, an average within-subject SD is reported. The slopes of the psychometric functions for each SRT measurement are estimated post hoc with nonlinear regression using iterative least squares estimation and a logistic function. To investigate the effect of training throughout the course of a test session, mean SRTs, as well as SD, are reported as a function of presentation order. The training effect was also assessed by fitting a linear mixed-effects model to the SRT estimates. The model included a fixed term for the presentation order, a fixed intercept term, and a random intercept term for each participant. An analysis of variance (ANOVA) marginal test (*F*-test) was then conducted to determine if the coefficients representing the fixed-effect terms were zero with a significance level of α=0.05. As a starting point, SRT estimates from both training and testing were included. The analysis was then repeated without the training.

## 3. Results

### 3.1. List Equivalence

**Figure 1:**
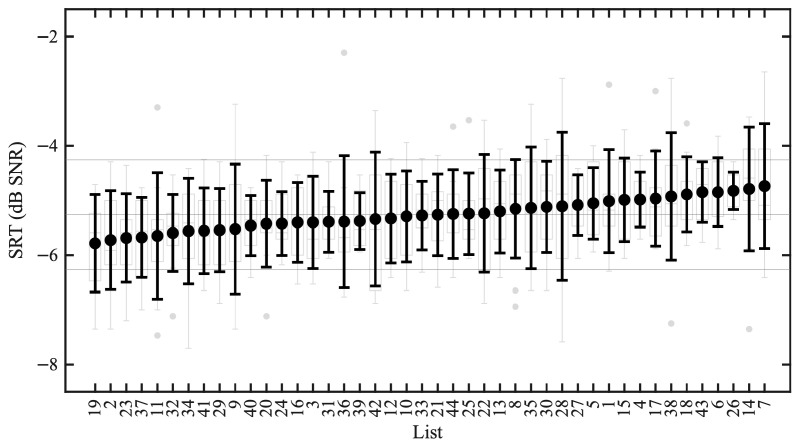
SRT distributions as a function of list number, where the lists are sorted by their mean SRT. The means of the SRTs across participants are indicated in the prominent circles, and the black error bars correspond to ±1 standard deviation. The underlying boxplots depict the percentiles, range, and outliers of the individual SRT estimates. The grand average SRT is marked with a gray horizontal line, as well as the SRTs 1 dB above and below the grand average. List 21 is missing one data point.

Figure 1 shows SRTs as a function of list number, where each mean of the SRTs across participants is indicated as well as the SD. The grand average SRT was −5.3 dB SNR (SD 0.3 dB), with mean SRTs per list ranging between −5.8 dB SNR (List 19) and −4.7 dB SNR (List 7). This resulted in a difference of at most 1.0 dB between all lists; thus, all lists obtained a mean within 0.5 dB of the grand average. Within-list variability was highest for List 28 with an SD of 1.4 dB and lowest for List 26 with an SD of 0.3 dB, and the average within-list variability was 0.8 dB.

### 3.2. Within-Subject Variability

**Figure 2:**
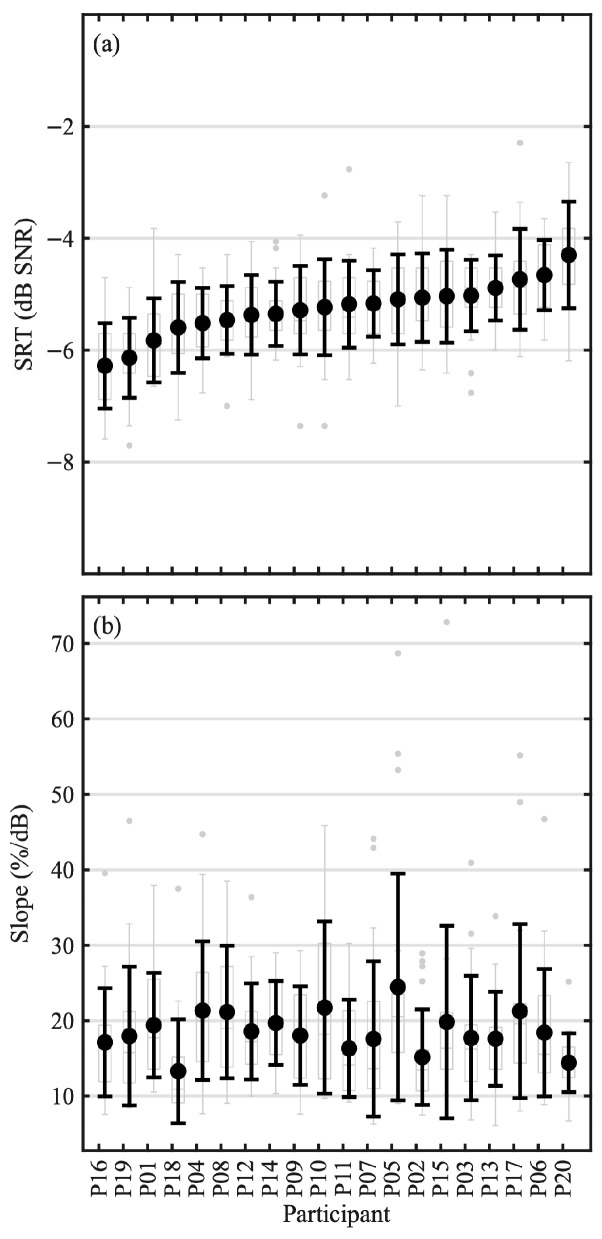
Distributions of the (**a**) estimated SRTs and (**b**) post hoc fitted slopes for each participant, where participants are sorted by their mean SRT. The means of the (**a**) SRTs and (**b**) slopes across lists are indicated in the prominent circles, and the black error bars correspond to ±1 standard deviation. The underlying boxplots depict the percentiles, range, and outliers of the individual estimates. P09 is missing one data point.

The distributions of SRTs obtained from each participant are shown in Figure 2a. Mean SRTs for individual participants ranged between −6.3 dB SNR (P16) and −4.3 dB SNR (P20) (grand average −5.3 dB with a SD of 0.5 dB). Within-subject SD of the SRT was on average 0.7 dB, with a range between 0.6 dB and1.0 dB. The distributions of the slopes from post hoc fitted psychometric functions are shown in Figure 2b. The mean of the slopes for each participant ranged between 13.3%/dB (P18) and 24.5%/dB (P05), with a grand average across participants of 18.5%/dB (SD 2.7%/dB).

### 3.3. Training Effect

**Figure 3:**
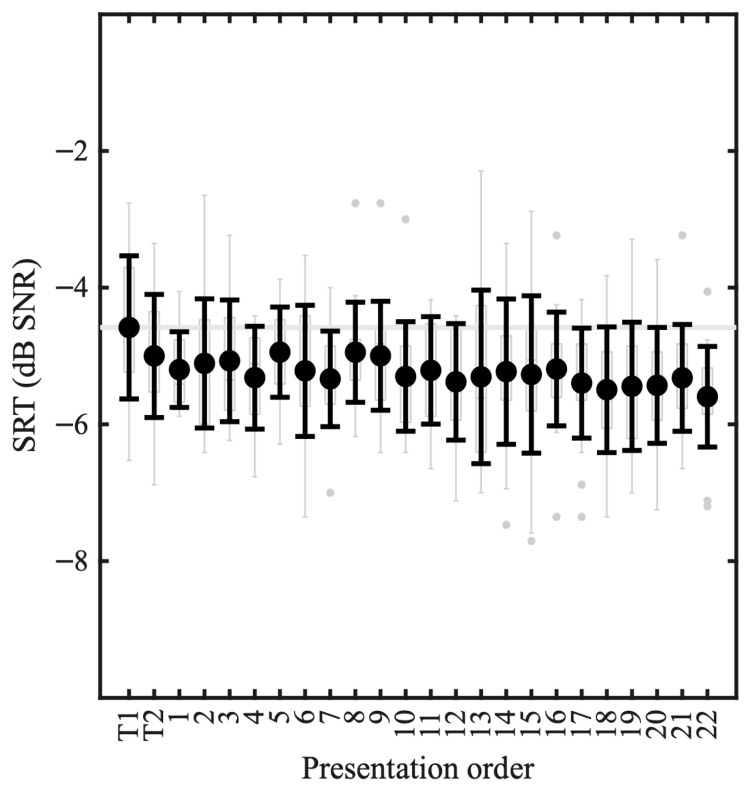
SRT distributions as a function of presentation order. The means of the SRTs across participants are indicated in the prominent circles, and the black error bars correspond to ±1 standard deviation. The underlying boxplots depict the percentiles, range, and outliers of the individual SRT estimates. The first two lists were training lists, and thereafter participants heard 22 test lists. Block 17 is missing one data point.

Figure 3 shows SRTs as a function of presentation order, where the means of the SRTs across participants—irrespective of the list presented—is indicated as well as the SD. There was a significant effect of the presentation order [$F\left(1,477\right)=21.4,\;p<0.001$] with an effect size (and 95% confidence intervals) of −0.023 dB/list [−0.03,−0.01]. When excluding the first two lists that were presented for training from the analysis, the presentation order remains significant [$F\left(1,437\right)=10.6,\;p=0.001$] with an effect size of −0.018 dB/list [−0.03,−0.01]. The mean SRT for the first training list was −4.6 dB SNR, the mean SRT after the 6th measurement was −5.3 dB SNR (i.e., a reduction of 0.7 dB), and the mean SRT for the 22nd measurement was −5.6 dB SNR (i.e., a reduction of 1.0 dB).

## 4. Discussion

The present study describes the development and validation of the Danish Sentence Test (DAST), a sentence-based speech-in-noise test designed to enable extended assessments of speech understanding using linguistically rich and variable sentence material without sentence repetition. Validation in this study was achieved by establishing normative SRT data for young, normal-hearing listeners and by demonstrating comparable performance across sentence lists when the test was administered under a defined headphone-based listening configuration using stationary speech-shaped noise and an adaptive procedure. The resulting normative mean SRTs, within-list and within-subject variability measures provide a reference for the use of DAST in research and development contexts that require repeated testing with large numbers of non-repeated sentence lists. In this context, the observed list-level deviations are small relative to the variability of individual SRT estimates, indicating that differences between lists fall within the expected measurement variability under the tested configuration. These normative estimates further allow the performance of DAST to be interpreted relative to earlier measurements obtained using non-adaptive procedures and related Danish speech materials. At the same time, these findings should be interpreted within the constraints of the specific listener population, playback setup, and scoring approach employed in the present study, and do not imply comprehensive validation across listener populations, experimental configurations, or clinical use cases.

Sentence-level RMS adjustment was applied in the construction of DAST in accordance with established recommendations for speech-in-noise test development (e.g., ICRA recommendations and ISO 8253-3) [19]. Such adjustments are widely used to reduce unintended variability in sentence intelligibility and to facilitate comparable measurement conditions across items and lists. Accordingly, the normative performance and list equivalence reported here should be interpreted as reflecting the behavior of DAST under standard, level-adjusted test conditions, rather than as properties of the unadjusted speech material in isolation. At the same time, applying sentence-specific level adjustments necessarily alters the distribution of sentence intelligibility relative to unadjusted conversational speech and may limit direct interpretations related to natural intelligibility variability in everyday listening environments, and thereby, ecological validity. Establishing test performance under alternative processing assumptions or without sentence-specific level adjustments would require separate empirical evaluation and remains outside the scope of the present study.

To place the normative performance of DAST in methodological context, the mean SRT of −5.3 dB SNR obtained in the present study—based on adaptive measurements collected from a separate listener cohort—is slightly more negative than the mean inflection point of −4.8 dB SNR reported previously for the full set of 1200 sentences when evaluated using a non-adaptive procedure [17]. While the optimization procedure was expected to reduce between-list variability, the present results demonstrate that this equivalence generalizes across measurement paradigms rather than being limited to the sentence-level data used during list construction. This difference aligns with expectations, as adaptive track procedures typically yield lower (i.e., better) SRT estimates than non-adaptive, fixed-SNR psychometric-function fits. Smits et al. [20] demonstrated that adaptive and non-adaptive SRT estimations can diverge due to differences in slope assumptions, response criteria, and the unequal weighting of sentence presentations across the adaptive track. Thus, the small discrepancy of approximately 0.5 dB between studies is consistent with the methodological differences in how SRTs were estimated rather than reflecting any inherent difference in the subset of speech material itself.

While DAST may be useful in many research and development applications requiring Danish language speech-in-noise assessments, it will not, and should not, replace all existing Danish corpora and tests. Each of these corpora and tests have their own purposes that will remain relevant. For example, Dantale I is one of the oldest Danish speech corpora still actively in use. It is a corpus consisting of audio and visual recordings of a female talker speaking monosyllabic words and digit triplets, and it was developed specifically for speech audiometry in Danish audiological clinics [4]. There is a long history with Dantale I in the clinic, and thereby a plethora of normative data, making it difficult to justify a change, even if one was warranted. Nonetheless, monosyllabic words and digit triplets are well-suited to these specific clinic tests, as the monosyllabic words and digits facilitate quickly estimating a discrimination score in quiet and a speech discrimination threshold, the latter of which is particularly useful for cross-checking air conduction audiometry. However, the test–retest variability of Dantale I has been shown to be relatively high, making it less suitable for applications where test–retest reliability is prioritized [21]. Outside of the clinic, one can also assess phoneme recognition using the Danish Nonsense Word Corpus (“Dansk NonsensOrdsKorpus” or DANOK), which was developed specifically for evaluating certain classes of signal processing algorithms that can affect phoneme perception due to potentially introducing changes to the spectral and temporal characteristics of the speech [22]. DAST is, at least in its current form, not specifically designed to investigate phoneme perception.

While word- and phoneme-based speech tests assess important aspects of speech perception, they are less suited for assessing a listener’s ability to follow natural forms of communication. Because each word is often presented in isolation, the words or phonemes often do not include many of the characteristics of natural speech, and as a result of this, it becomes difficult to assess more modern hearing aids, as the underlying adaptive algorithms do not necessarily work as intended in such unnatural listening scenarios (e.g., compression and noise reduction). Investigations of speech understanding, therefore, often employ sentence-based speech material instead. One example of such a test is the Danish sentence test Dantale II [9]. It is a matrix test that follows the same principles as those developed in other languages [7,8], where every list in the corpus consists of 10 different five-word sentences with the same syntactical structure: <Name> <verb> <numeral> <adjective> <object>. There are 10 names, 10 verbs, 10 numerals, 10 adjectives, and 10 nouns, giving a corpus of 50 words in total. The Dantale II material is routinely employed in two different tests: one where the test participant is not made aware that it is a closed set of vocabulary and a scorer assesses whether they correctly repeat the words, and one where the test participant is asked to select the five words in the sentence via an interface that reveals the matrix format (i.e., multiple choice). In either case, the level of the speech relative to the noise is adapted depending on the listener’s earlier responses to obtain an SRT estimate in a similar way to DAST. The consistent structure of the sentences in this speech material gives a higher level of control than DAST, meaning it still may be the right choice in applications where such control is advantageous. Additionally, the fact that similar matrix sentence tests exist in many other languages around the world will make investigations with Dantale II more comparable and repeatable than DAST.

HINT provides an alternative approach to matrix sentence testing as it consists of sentences that better represent conversational speech [11]. Based on the principles of HINT, Nielsen & Dau [23] developed the conversational language understanding evaluation (CLUE) test for the Danish language, and then subsequently developed a standardized Danish version of HINT based on the same speech material. The primary differences between the Danish HINT and CLUE relate to which sentences are included in the formalized lists, to how scoring is conducted, and to how the adaptive track is adjusted to estimate an SRT. In comparison to HINT, DAST provides the substantial advantage that it comes with more than four times the number of lists. However, like the matrix sentence test, HINT adaptations also exist in many other languages around the world, meaning it may still be advantageous to choose HINT over DAST if transferability is desirable.

One area of interest in the study of speech understanding has been to measure a listener’s ability to recognize speech in the presence of interfering speech (i.e., speech-on-speech masking), and moreover, to assess how this can change when the target and interfering speech are spatially separated [24,25]. These aspects are particularly interesting due to their potential connection to listening in more complex listening environments. For such experiments, researchers have traditionally used speech material that is syntactically similar across sentences in order to increase the similarity between the target and interfering speech, and thereby, increase the difficulty of the segregation task. Dantale II sentences have been a popular choice for this in Danish, as well as the Danish open-set, low-context “DAT” corpus [26]. The sentences in the DAT corpus start with one of the female names Dagmar, Asta, or Tine and are then followed by a carrier sentence that ends with two target keywords, which are essentially unpredictable: “<Navn> tænkte på <substantiv> og <substantiv> i går” which translates to “<Name> thought about <noun> and <noun> yesterday”). The keywords are temporally aligned to maximize the mutual masking. The task of the listener is to repeat only the two keywords. A specific version of the corpus has been employed to make a test that is suitable for school-age children [27]. The concept of the DAT corpus is inspired by the coordinate response measure (CRM) corpus [28], which is a popular English speech corpus that consists of sentences in the form: “Ready <name>, go to <color> <number> now.” In these sentences, the <color> and <number> can be one of 32 color-digit combinations created from four colors and the digits one to eight, and the <name> can be any one of eight possible names that make up the call sign to cue the listener of the target sentence among other interferring sentences. As with the matrix sentence tests, however, the DAT and CRM sentences do not represent well the variation that is present in everyday, conversational language, and therefore, are most well-suited for investigations that benefit from similarity across sentences. For this reason, the Danish HINT has also been applied in a competing-voices paradigm [29], and following this, DAST could be applied as well.

It has been argued that the fixed syntactical structure of a matrix sentence test increases predictability, which contributes to the relatively large reported learning effect: mean SRTs dropped 3.2 dB from the first to sixth SRT measurement with Dantale II [10]. In comparison, the within-visit improvement after the sixth SRT measurement in the current study was 0.7 dB, increasing to 1.0 dB after 22 measurements—a magnitude that remains relevant for experimental design and interpretation in speech-in-noise testing. In our statistical analysis, it is estimated that the SRT estimate drops approximately 0.02 dB per measurement. For reference, the Danish HINT has shown learning on the order of 0.5–0.8 dB across repeated exposures [30], suggesting that differences in linguistic structure and predictability across sentence materials may contribute to differences in observed training effects; however, potential training effects across sessions remain unexplored. These results underscore the importance of training lists and appropriate list counterbalancing when repeated speech-in-noise measurements are collected within a session, particularly in studies targeting small effect sizes.

While the primary objective at the outset of this project was to create a Danish-language speech-in-noise test with more speech material than the Danish HINT, a secondary objective was to create a speech-in-noise test intended to yield SRT estimates that would more closely reflect real-world conversational speech than existing tests. By creating longer sentences with more complex vocabulary and linguistic syntax than the Danish HINT, we anticipated that DAST would thereby provide more positive SRT estimates. However, the results of the current study suggest that this is not the case, as mean SRTs for the NH participants were −5.3 dB SNR with DAST, but −2.5 dB SNR with HINT [12], and for reference, −8.4 dB SNR with Dantale II [9]. We have, nevertheless, shown in an earlier study that employing HINT-like scoring methods with the DAST material leads to SRT estimates similar to those of HINT [17]; in particular, scoring a sentence as either correct or incorrect as opposed to using more scoring units per sentence seems to be a key element to increasing the resulting SRT estimates. This aligns with earlier work showing that the scoring method can play a substantial role in determining the resulting SRTs of a test [31]. Taken together, these observations suggest that the choice of scoring method can substantially influence resulting SRT estimates, particularly in sentence-based tests that employ more conversational speech material with an open set of vocabulary.

For context though, current estimates of the SNRs that are typically encountered in the real world are quoted as being higher than HINT SRTs, i.e., on the order of 0 dB SNR to +5 dB SNR [32] and −2.5 dB SNR to −0.5 dB SNR [33]. Importantly though, these numbers reflect the range that is typically encountered, but they do not necessarily reflect the entire range of SNRs that are experienced, as many listeners in these studies did in fact encounter more negative SNRs, albeit less often. Therefore, it may be an ill-posed objective to design an SRT-based test that operates in the range of SNRs people typically encounter in everyday life, as the SNRs people typically choose to be in may, at least in part, be driven by the desire to be able to understand more than 50% of the speech. Thus, the SRTs we measure with speech-in-noise tests may in fact be realistic SNRs, but nevertheless atypical.

## 5. Conclusions

This study described the development and validation of the Danish Sentence Test (DAST), a sentence-based speech-in-noise test designed to enable extended measurements using linguistically rich sentence material without repetition. The test consists of 44 balanced lists of 20 sentences each, which are available for download (https://doi.org/10.11583/DTU.24058110) together with the purpose-built software (https://github.com/abbiekressner/dast-app, (accessed on 11 May 2026)). Validation in the present work consisted of establishing normative SRT data for young, normal-hearing listeners and demonstrating comparable performance across sentence lists when the test was administered under a defined headphone-based listening configuration with stationary speech-shaped noise and an adaptive procedure.

Under these test conditions, mean SRTs of −5.3 dB SNR were obtained, with mean list-level deviations within ±0.5 dB of the grand average, relatively small within-subject variability, and an estimated average psychometric slope of 18.5%/dB. Together, these results indicate that the sentence lists yield comparable performance outcomes under the tested paradigm and provide a normative reference for the use of DAST in research and development contexts requiring repeated measurements with non-repeated sentence material. It is important to note, however, that the present findings do not constitute a comprehensive evaluation of test performance across listener populations, playback configurations, or scoring paradigms. Additional work is required to establish test–retest and inter-session reliability, sensitivity, and normative performance in hearing-impaired listeners, older populations, and alternative experimental setups commonly used in clinical or applied contexts.

DAST complements the existing set of Danish-language speech-in-noise tests and provides an additional option to consider when designing listener studies. It may be a particularly attractive option for investigations requiring extensive speech-in-noise measurements using more linguistically complex sentence material. More broadly, its design philosophy—balancing linguistic richness with psychometric control—illustrates an approach that may inform the development of sentence-based speech-in-noise tests in other languages.

## Figures and Tables

**Table 1:** Example list (i.e., List 1). The keywords in each sentence are underlined.

Sentence	Template	Offset (dB)	Words	Translation
1134	4	−0.8	Jeg indså hvad de havde baseret deres overskud på	I realized what they had based their profit on
159	9	+2.7	Det er kun pressen som anvender meningsmålingen	It is only the press that uses the opinion poll
518	8	+2.2	De har røbet at de snart vil øge deres støtte	They have revealed that they will soon increase their support
312	2	−0.7	Han er heldig fordi lægen vil lave en henvisning	He is lucky because the doctor will make a referral
1062	2	+0.6	Vi er naturligvis utilfredse fordi I har afbrudt festivalen	We are naturally dissatisfied because you have interrupted the festival
253	3	−0.4	En eller anden journalist ventede på mig i køkkenet	Some journalist was waiting for me in the kitchen
955	5	−0.8	Det er imponerende at I engagerer jer så meget i politik	It is impressive that you engage so much in politics
908	8	−1.8	De insisterede på at vi skulle udbetale vores gæld	They insisted that we should pay off our debt
496	6	−0.2	I efteråret vil de producere endnu flere møbler	In the autumn they will produce even more furniture
677	7	−0.6	Hun kan lige dække omkostningerne med sin løn	She can just cover the expenses with her salary
121	1	+0.3	Der var nogle eksperter der blandede sig i debatten	There were some experts who intervened in the debate
100	10	−1.2	Vi opfordrer folk til at ringe til vores talsmand	We encourage people to call our spokesperson
951	1	+0.1	Der var en rytter der satsede alt på første etape	There was a rider who gambled everything on the first stage
564	4	+0.3	De har skrevet hvor længe nummeret vil vare	They have written how long the number will last
667	7	+0.4	Jeres repræsentant har oplyst os om vilkårene	Your representative has informed us about the terms
723	3	+0.1	Han hævede stemningen med sine kommentarer	He lifted the mood with his comments
1069	9	−2.1	Det var en talsmand som udsendte den her besked	It was a spokesperson who sent out this message
1120	10	+1.6	Jeg vil modernisere både produktionen og leveringen	I want to modernize both the production and the delivery
255	5	+0.8	Det var vanskeligt for ham at parkere vognen	It was difficult for him to park the wagon
326	6	+0.3	I den her sæson må vi nok opgive håndbold	In this season we will probably have to give up handball

## Data Availability

The data presented in this study are openly available at https://doi.org/10.11583/DTU.24058110.
